# How we define recurrent miscarriage matters: A qualitative exploration of the views of people with professional or lived experience

**DOI:** 10.1111/hex.13607

**Published:** 2022-09-26

**Authors:** Rebecca Dennehy, Marita Hennessy, Sarah Meaney, Karen Matvienko‐Sikar, Ria O'Sullivan‐Lago, Jennifer Uí Dhubhgain, Con Lucey, Keelin O'Donoghue

**Affiliations:** ^1^ Pregnancy Loss Research Group, Department of Obstetrics and Gynaecology University College Cork Cork Ireland; ^2^ INFANT Research Centre University College Cork Cork Ireland; ^3^ National Perinatal Epidemiology Centre University College Cork Cork Ireland; ^4^ School of Public Health University College Cork Cork Ireland; ^5^ Waterstone Clinic Cork Ireland; ^6^ RE:CURRENT Research Advisory Group, Pregnancy Loss Research Group, Department of Obstetrics and Gynaecology University College Cork Cork Ireland; ^7^ Miscarriage Association of Ireland Carmichael Centre Dublin Ireland

**Keywords:** early pregnancy loss, interviews, miscarriage, qualitative research, recurrent miscarriage

## Abstract

**Background:**

Recurrent miscarriage (RM) affects 1%–3% of women/couples of reproductive age depending on the definition used, for example, whether 2 or ≥3 miscarriages. Stakeholders' views of how RM is defined have received limited attention to date. A definition reflects the medical evidence and values of a society at the time, and thus warrants ongoing review.

**Aim:**

We aimed to explore the views of women and men with lived experience of RM, and those involved in the delivery/management of services and supports, on how RM is and/or should be defined.

**Methods:**

We adopted a qualitative study design, incorporating semi‐structured interviews. We used purposive sampling to recruit participants in the Republic of Ireland, ensuring diverse perspectives were included. Women and men with lived experience of ≥2 consecutive first‐trimester miscarriages were recruited via health professionals and social media; other participants via the research team's networks. Interviews were audio‐recorded, transcribed, pseudo‐anonymized and analysed using reflexive thematic analysis.

**Findings:**

We conducted interviews with 42 health professionals/service providers and 13 women and 7 men with lived experience of RM (June 2020 to February 2021). We generated three interrelated themes from the data: (i) *The need for a standardized definition of recurrent miscarriage–Finding a balance between research evidence, individual needs and healthcare resources*, (ii) *The definition is a route to finding an answer and/or validating women/couples' experience of loss* and (iii) *Working around the definition—Advocacy and impacts*.

**Conclusion:**

A nuanced approach to defining RM is warranted, one which is evidence‐informed recognizes the individual needs of women/couples, and considers healthcare resources.

**Patient or Public Contribution:**

Members of the multidisciplinary RE:CURRENT (REcurrent miscarriage: evaluating CURRENT services) Project Research Advisory Group (including four parent advocates, two of whom are co‐authors on this article) were actively involved throughout the study, including the generation of topic guides and the refining of themes.

## 1 INTRODUCTION

Recurrent miscarriage (RM), also referred to as recurrent pregnancy loss, is a much‐debated condition that affects 1%–3% of women/couples of reproductive age depending on the definition applied.^1^ International guidelines vary in the term used and how it is defined.^2^ For example, the American Society for Reproductive Medicine uses the term ‘recurrent pregnancy loss’ and defines it as the loss of two or more consecutive pregnancies, both of which must be confirmed by ultrasonography or histopathology.^3^ The Royal College of Obstetricians and Gynaecologists (RCOG) adopts ‘recurrent miscarriage’, which it defines as three or more first‐trimester miscarriages (which do not have to be consecutive, or with the same partner); however, they encourage clinicians to conduct investigations after two first‐trimester miscarriages based on an individualized approach.^4^ In the Republic of Ireland, RM is generally defined as three or more (consecutive) miscarriages.^5^ The challenge in achieving consensus regarding the definition was noted by the European Society of Human Reproduction and Embryology (ESHRE) who suggested a definition of two or more miscarriages (irrespective of whether consecutive or not) but acknowledged that their members did not all agree on this matter.^6^ Research is needed to inform the development of consensus on the definition of RM, including when necessary investigations should be undertaken.^2^

Establishing the appropriate investigations for women/couples after one, two, three or more miscarriages is a priority for miscarriage research.^7^ Van Dijk et al.^8^ concluded that investigations could be conducted after two (rather than three or more) pregnancy losses, but cautioned the need for further research on the prognostic value of test results. In their retrospective cohort study, Youssef et al.^9^ found no difference in the distribution of associated factors in couples with two versus three or more pregnancy losses. More recently, Coomarasamy et al.^10^ proposed a graded approach to miscarriage care in an attempt to balance the need for evidence‐based management and supportive care, and resources. They suggest women/couples are offered support following one miscarriage, care in a nurse or midwifery‐led clinic with some investigations following two miscarriages and care in a consultant‐led clinic with comprehensive investigations following a third miscarriage.

Notwithstanding the importance of the application of clinical guidelines for RM, their use may result in a probable diagnosis for only half of women/couples.^6^, ^11^ Additional tests, such as the addition of 24‐chromosome microarray on pregnancy tissue, alongside standard tests, may provide more explanations^11^; however, this is not without resource implications. In practice, care provision can deviate from clinical guideline recommendations, with greater maternal age and a lower number of preceding miscarriages (i.e., two rather than three) guiding when investigations are conducted.^12^, ^13^ In their study concerning the application of the RCOG guideline, Manning et al.^13^ found that, while most clinicians investigated women with ≥3 consecutive pregnancy losses (79%), some also reported investigating ≥3 nonconsecutive losses (22%), and ≥2 consecutive (22%) and nonconsecutive (6%) losses. Public perceptions of how RM is defined can also vary, with two miscarriages (consecutive/not) being the most commonly cited in a nationally representative Irish study.^14^

There has been limited stakeholder involvement in the development of clinical guidelines for RM, and in related discussions about how the condition is defined.^2^ Furthermore, while some research has examined the lived/care experiences of women and men with RM,^15^, ^16^, ^17^ how the condition is defined has received limited attention. Women and their partners have expressed frustration when denied investigations following a second miscarriage, particularly in cases of advanced maternal age.^16^, ^17^ In addition, they have expressed frustration around the lack of recognition of the psychological impacts of RM.^17^ Many who experience RM can feel powerless. Women/couples often attend many different healthcare professionals/clinics searching for a cause and treatment^10^ as searching for answers can help them feel in control.^17^ An important aspect of RM care is providing individualized care without normalizing or over‐medicalizing the condition.^10^, ^18^

Terms such as RM are ‘more than just words: they carry with them a range of meanings and social consequences’.^19^ Defining/diagnosing a condition can facilitate access to care, including investigations, and legitimize lived experiences; it can also grant power to clinicians to allocate resources and labels.^20^ A definition reflects the medical evidence and values of a society at the time,^19^ thus warrants ongoing scrutiny and potential revision.

The aim of this study is to explore views of how RM is and/or should be defined from the perspectives of women and men with lived/living experience of RM and those involved in the delivery/management of services and supports. This study question is set within the context of a broader study investigating views on RM services and supports in the Republic of Ireland, where there is currently no national clinical guideline specifically for RM care.^5^ Exploring the views of women and men with lived experience of RM, and those working in the area is needed so that appropriate care can be given; how we define RM is where such care begins.

## 2 METHODS

We utilized a qualitative study design underpinned by social constructionism which acknowledges that knowledge and meaning are socially produced.^21^ We undertook semi‐structured interviews to facilitate an in‐depth exploration of views regarding the research aims, acknowledging the active role we played in co‐creating knowledge and meaning. The multidisciplinary research team included a woman and man with lived experience of RM (J. U. D. and C. L.), three social scientists (R. D., S. M. and R. O. S.‐L.), a public health/health services researcher (M. H.), a health psychologist (K. M.‐S.) and an obstetrician and maternal–foetal medicine subspecialist (K. O. D.). The study is reported in line with the Standards for Reporting Qualitative Research (see Supporting Information: File).^22^

### 2.1 Ethical approval

Ethical approval for the study was granted by the Clinical Research Ethics Committee of the Cork Teaching Hospitals [Reference number: ECM 4 (ff) 10/03/2020 & ECM 3 (gggg) 09/04/2020].

### 2.2 Setting and participants

We purposively sampled individuals involved in the delivery/management of RM services and supports in the Republic of Ireland and women and men/partners with lived experience of RM. We aimed to ensure perspectives from different geographical areas, settings (hospitals/clinics and primary/community care), hospital groups, types/sizes of maternity units or hospitals (tertiary, regional and peripheral), and types of RM services (dedicated RM or pregnancy loss clinics, and nondedicated services within broader gynaecology services) in the Republic of Ireland were included. Women and men with lived experience of two or more consecutive miscarriages (most recent miscarriage 6–24 months before participation) were recruited via health professionals and social media. Individuals involved in the delivery/management of RM services and support were recruited via the research team's networks, including project collaborators.

### 2.3 Data collection

Participant views were explored using semistructured interviews, conducted by RD and MH via Zoom^23^ between June 2020 and February 2021. Participants were asked to provide a phone number so that the interview could be completed by phone in the event of a poor or dropped internet connection.^24^ Participants provided informed, electronic consent in advance of the interview; ongoing verbal consent was also sought.^25^ Topic guides were used to frame the discussion: one specific to women and men who had experienced RM, and another tailored for healthcare professionals (see Table 1 for an overview of topics). We used the concept of ‘information power’^26^ to determine when to cease sampling; stopping when the major categories showed depth and variation—both within and across interviews with people with lived experience of RM, and those involved in the delivery/management of services and supports. Interviews were audio‐recorded, and interviewers took detailed field notes following each interview.

**Table 1:** Topic guide structure

Health professionals	Women and men with lived experience of recurrent miscarriage
Defining recurrent miscarriage	Expectations of pregnancy before loss
Structure of care	Management of recurrent miscarriage
Management of recurrent miscarriage	Subsequent pregnancy
Knowledge and understanding	Knowledge and understanding
Impact and support	Impact and support
Recommendations	Recommendations

### 2.4 Data analysis

The audio files were transcribed verbatim by a professional transcriber.^27^ Transcripts were checked, de‐identified/pseudo‐anonymized, and imported into NVivo 12^28^ for data management. We analysed the data using reflexive thematic analysis, recognizing the socially produced nature and meaning of language and discourse in line with social constructionism,^21^ using a data‐driven inductive approach. Patterns (i.e., themes) were identified through a rigorous process of data familiarization, data coding, theme development and revision.^21^ We analysed data from women and men with lived experience and those involved in the delivery/management of services and supports collectively to provide a multifaceted understanding of how RM is defined.

All members of the research team were involved in the analysis which was led by R. D. and M. H., with R. D., M. H, S. M., K. M.‐S. and R. O. S.‐L. meeting regularly to discuss the transcripts, preliminary codes and categories and examine their positionality in relation to these. Themes were reviewed and discussed at a meeting of the RE:CURRENT Project Research Advisory Group, with subsequent further discussions with J. U. D. and C. L.

## 3 RESULTS

Between June 2020 and February 2021, we conducted interviews with 42 health professionals/service providers and 13 women and 7 men with lived experienced RM (see the breakdown in Table 2). The 20 women and men had experienced a range of first‐trimester miscarriages (0–15) including numbers of consecutive miscarriages. Fourteen had living children and three were pregnant at the time of the interview. Two of the participants with lived experience were married couples; they were interviewed separately. Interviews lasted 18–119 (average: 50) min for health professionals/service providers, and 25–98 (average: 117) and 21–56 (average: 36) min, for women and men who experienced RM, respectively.

**Table 2:** Overview of participants by stakeholder group

Stakeholder group (identifier)	Number of participants
Clinical Midwife/Nurse Specialists in Bereavement and Loss (CMS)	8
Consultant Obstetricians/Gynaecologists (Hospital‐based) (OBGYN‐H)	4
Consultant Obstetrician (Private fertility sector) (OBGYN‐FC)	1
Specialist Registrars (SPR)	2
Nurses (GN), Midwives (M), Sonographers (S)	4
Chaplaincy and Pastoral Care (CP)	2
Support Services (Perinatal Mental Health (PMH), Social Work (SW), Community and Voluntary (VS)	3
GPs (GP)	4
Practice Nurses (PN)	2
Public Health Nurses (PHN)	2
Administrative Support (AS)	1
Maternity Hospital/Unit/Group Level Administration, Governance and Management—Directors of Midwifery (DOM); Group Director of Midwifery (GDOM)	3
National Administration, Governance and Management (AGM)	6
Women who have experienced recurrent miscarriage (PW)	
Age range: 32–44 years (*n* = 12^a^; mean age: 39 years)	13
No. of first‐trimester miscarriages^b^: 2 (*n* = 4); 4 (*n* = 2); 5 (*n* = 1); 6 (*n* = 3; one of which had two twin pregnancy losses); 7 (*n* = 1); 9 (*n* = 1); 15 (*n* = 1)	
Men who have experienced recurrent miscarriage (PM)	
Age range: 28–41 years (*n* = 6^a^; mean age: 34 years)	7
No. of first‐trimester miscarriages: 2 (*n* = 2), 3 (*n* = 3), 5 (*n* = 1), 9 (*n* = 1)	
Total	62

^a^ One participant did not disclose their age.

^b^ Two participants also experienced additional losses: second trimester loss/stillbirth/infant death.

We generated three interrelated themes from the data which detail how those with professional and lived experience conceptualize how RM is defined/operationalized: (i) *The need for a standardized definition of RM – Finding a balance between research evidence, individual needs and healthcare resources*, (ii) *The definition is a route to finding an answer, validating women/couples' experience of loss and providing necessary supports* and (iii) *Working around the definition—Advocacy and impacts*. These themes, and their subthemes, with illustrative quotes, are outlined in Tables 3, 4, 5.

**Table 3:** THEME 1: The need for a standardized definition of recurrent miscarriage– Finding a balance between research evidence, individual needs and healthcare resources: Subthemes and illustrative quotes

Subtheme	Illustrative quote	Quote no.
The need for standardization in practice	I think it will certainly be useful for us as clinicians in Ireland to have somewhat of a consensus I think of what we define as recurrent pregnancy loss, and what investigations and all of that, just to have some kind of uniformity rather than somebody going to this hospital and having this test done, and then going to another hospital and being denied that test. … I think it would be useful for clinicians as well as patients… (OBGYN‐H1)	1.1
Following the evidence: Who benefits from investigation and treatment/intervention	I think, it seems quite cruel if you've got someone you know sitting in front of you who's just got this devastating news, and you're saying no sorry you can't go to the clinic because you don't meet the criteria. But I suppose, you know, we need to prioritise appointments in the clinic for the people who could benefit the most from it, rather than giving it to everyone. And also the chances are if they don't meet the criteria, you know, that there isn't going to be any benefit from going to the clinic and putting all the investment, both financially and emotionally, in these tests that probably won't prove much benefit for them. They probably will have a successful pregnancy the next time themselves without any intervention. (OBGYN‐H4)	1.2
Acknowledging complexity and need for flexibility: Considerations beyond the number of miscarriages	It's a difficult one because … you'll have a lot of women who'll have two miscarriages and then go on to have a successful pregnancy. But again, I think it depends on age. You know if you've a woman over 35 who's had their second miscarriage, I think it's reasonable to refer them on and see if there's anything that can be done … rather than just waiting for another one you know. (GP4)	1.3
	…the RM clinic is very much, has got you know direct input from the consultant in charge of it, and they are quite formidable so you wouldn't add someone to their clinic without their approval unless they met the strict criteria. (OBGYN‐H4)	1.4
	…to go through all that even twice, I think it's heart wrenching for women. And to put them through it a third time. Okay, I know there's evidence, and there's research, and, you know, but I think empathy and compassion has to have a place here for those women … this is a baby to them. This is not a piece of tissue… (AGM2)	1.5
	…if you have two miscarriages together, I think that should be enough to investigate. I think the third one puts a big, huge mental block on the woman, you know, because, as I said, you're almost waiting for it. Like you're saying like, I'll be really lucky if this works, but you're basically thinking, you know, like I think you get stuck in that cycle anyway of miscarriage and pregnancy, so you're almost expecting it. I think there's pressure then to have to wait for that third one… (PW9)	1.6
	I hate the tick box world that we're living in saying ‘well have you met this this this and this’ but actually to consider and also give them the support that if they are just really anxious that they also need that support to assist them in perhaps conceiving naturally and just reassure them … I would say there is that criteria of, you know, three and that's really for staff on the ward who are seeing them coming in for miscarriages and in the early pregnancy assessment. But actually, it is taken really on the individual need. But I think there is a gap there do you get me? (CMS3)	1.7
	It's come up in a few meetings I suppose where people would have gone into the hospital having had their third miscarriage but having had a child in the middle to be told oh we can't do the investigations because it's three in a row is what we look for. And it just seems a bit barbaric to them that they're being told even though you've had three you actually have to have two more after this before it will be looked into. (S1)	1.8
	Why would you wait for three dead babies, and three dead babies in a row. God forbid you have a live baby in between like you were back to square one. It's stupid. Absolutely stupid. You know, if your arm kept going dead, they'd check it… (PW3)	1.9
	I went in [to hospital] for four and I had some at home because they were earlier miscarriages, so you know, I was able to manage it myself at home. But then of course you're in a situation where they don't count that. So, I think that's a huge problem for women. You know, even when I went in now with the recent miscarriage and they were saying oh, this is your fourth miscarriage, and I was saying well actually no, it's my seventh … You know, I just couldn't get over that a midwife was arguing with me about the number of losses that I had experienced knowing I had had seven. (PW1)	1.10
Limited resources constrain how recurrent miscarriage is defined and/or how the definition is operationalized	…it would often be the basis of complaint you know that they weren't given an appointment to the RM clinic you know. And you know at the time it [investigating after three miscarriages] was to do with, if my memory can serve me, it was to do with resources really you know. The clinic was only run every two weeks by the consultant, and I suppose there was only one consultant doing it. And you know it came down to that really the resources to it had at the time only one clinical midwife specialist as well who was doing all the counselling you know for bereavement as well as everything else. (AGM3)	1.11
	Just making sure like from a medical point of view that they do offer something just to check your general health … I know its cost and time and resources and that's always gonna be the answer to push back on these things. But I don't know what analysis you guys are doing you know to show the business case of why these things should be done but surely they need to. And from just a point of like respecting women and enabling women to learn more about their bodies I think the services could provide more information and maybe just something that doesn't make you feel like it's not significant … Maybe more information after the first one to prepare you that there could be a second or a third. And then that in between bit. I mean it's so anxious when you've had a second. I make jokes I'm like am I gonna have a hat trick. (PW13)	1.12

**Table 4:** THEME 2: The definition is a route to finding an answer, validating women/couples' experience of loss and providing necessary supports: Subthemes and illustrative quotes

Subtheme	Illustrative quote	Quote no.
Looking for a reason/answer	I think once people have had two consecutive miscarriages, they kind of want answers. So it's quite hard to reassure someone that they should try again and the whole emotion of trying to go through another miscarriage before they are eligible for any investigations, particularly when there's an age thing on it you know depending on somebody's age. The timeframe might be an important factor for them as well. (S1)	2.1
	So, the test was done. So, we had the results. So that's why I'm quite emotional because the results just came back last week. *Cries* That we would have a healthy girl if everything went fine. Nothing wrong. If the pregnancy was healthy. So, it was me. It's my body doing it. It's killing it. … Because I was hoping because if there is a genetic problem at least we have the answer. But then when it came back that the baby would be healthy… (PW6)	2.2
	It's one of the most important things of looking after women is to set the expectations realistically. And it's a line I will often use. When it comes to pregnancy there are no guarantees. So, I as your doctor can do all the investigations. I usually will not find a cause. In circumstances that I find a cause the treatment may not work. In circumstances where I find a cause and there is a good treatment, I can't guarantee the treatment will work because you could miscarry for another reason. (AGM5)	2.3
Feeling frustrated, dismissed when you don't meet the criteria	Yeah it's not a good feeling [to be refused referral/investigation after 2]. After the first one and the second one it's kind of I suppose I felt very frustrated and just kind of completely helpless as well. Because it's something that we really wanted. It something that we were absolutely devastated about. And being kind of told, well, look just, you know, dust yourselves off and go for it again … But really just kind of from my own mindset, it's very hard to try and contemplate going through that process again … if it was something that was diagnosable and something that was preventable or something that was treatable you know we'd be in a much better I think headspace to go for it again. (PM5)	2.4
	But it's very sad because a lot of the women would after two and the question is ‘why do I have to have three’, you know, ‘why do I have to have three’. And you're trying to explain to them about how you know the statistics are that actually an awful lot of us have one and then a lot less of us still have two. And it's very hard explaining that to a woman who is grieving. And the difference between a recurrent lady I think and to a lady that's had a one is that there's this whole heap of anxiety much more so… (CMS7)	2.5
Existing in a liminal space, falling between the cracks in service provision	…is it two miscarriages or three miscarriages? Should it be the women of two miscarriages that are getting this, because there is a gap between the two and the three from my experience where the women are outside the service. They're outside us assisting them you know so they are struggling out there on their own unless they're navigating it themselves through their GP or whatever. (AGM3)	2.6
	Our definition of RM … would be three successive miscarriages in a row. Like we have a lot of women coming who would have had one miscarriage or two miscarriages, and they've never got to the RM clinic. Those women I deal a lot with every day, and they do need a lot of emotional support. (M1)	2.7

**Table 5:** THEME 3: Working around the definition—Advocacy and impacts: Subthemes and illustrative quotes

Subtheme	Illustrative quote	Quote no.
Advocacy efforts: ‘To hell with the system’, ‘Just lie and say you've had three’ [Over‐riding the referral criteria]	I suppose for me, my thing is, I'm torn. I see somebody who is gonna have to wait for a third miscarriage and again sometimes you might engineer their appointment coming back for when someone else is on call … to get them over the line yeah or to kind of maybe get their investigations a little bit earlier (S2)	3.1
	We were upset [about being denied investigations/referral after 2] but if it wasn't for *Consultant* you know we would be waiting another round. And the nursing staff they were brilliant. (PM2)	3.2
	You might start lying and lying and saying I've had three and then you're just in that thing where you start making things up to get what you want. And sure, what's the point in that. Yeah. And then you're gonna be in the system so you have to lie or do something dishonest to get yourself in the system. But then the real reason you're doing that is because you're scared, and you're terrified, and you don't know what's going on inside your own body. And it's like the medical services have the power over whether you get to know if there's something wrong with your body or not. And you're given a number. I understand they have to manage things and statistics. Maybe three is the number that they say right any more than three warrants investigation but the amount of time a woman loses in her life, it could be like a year or another year. (PW13)	3.3
	Oh my God that anxiety when they're pregnant again is horrendous, you know. You will see a lot of them in that pregnancy if everything goes to plan but if it's a miscarriage it's so devastating. And you know you nearly have people coming in and telling you oh I had positive tests at home, I've had a really heavy bleed, its negative now, I've had another miscarriage. And sometimes you'd wonder are they just telling you that so that they can be investigated …. And then some consultants will say well no we don't have evidence of that. And I just think oh my gosh, that's terrible. You feel like you're making a liar out of the woman. (S2)	3.4
Dealing with inappropriate referrals: ‘They still send in people which is really cruel’	See quote within text	N/A
‘If it's not available in the public they will go to the private’	Well patients who are in this position are desperate, and desperate people will do anything to get the result. So, they are a group that could be manipulated. I mean it's the same with infertility and IVF. Families are absolutely desperate to get a pregnancy. They'll do anything. They'll pay anything. And this would be another group that could be manipulated. And clearly that has got to be avoided. And the temptation from the public's point of view is that if you have RM and some therapy is providing you have a pregnancy then you automatically attribute the pregnancy to the therapeutic intervention whereas in fact it could have happened without any intervention. (AGM4)	3.5
	I don't know if we're even supposed to be telling them about the private service, but I do, because I can't bear it like. You know I say to them, you know, you can go private. And I tell them to just ring *Hospital D* because I know we're not supposed to be giving names and stuff. And I just say look get all of your options. There are options, you know. And yeah, they will then. A lot of them will go private. Because when I used to coordinate the miscarriage clinic often when you were ringing them for their appointments, they've already gone private. (CMS7)	3.6
	I was seeing people in *Fertility Clinic 5* for fertility and assessments and doing IVF. We would often, funnily, sometimes you would get referrals in, somebody has had two miscarriages and is really worried, will you see them in *Fertility Clinic 5*. So, I wouldn't see them publicly, and then they send the same letter in, will you see her privately. *Laughs* And I go, ‘absolutely not’. (OBGYN‐H2)	3.7

### 3.1 THEME 1: The need for a standardized definition of recurrent miscarriage: Finding a balance between research evidence, individual needs and healthcare resources

This theme describes how defining RM—and operationalizing that RM definition as referral criteria for services—involves finding a balance between the research evidence, needs of women/couples with RM, and available healthcare resources, all of which can be a complex endeavour. In practice, RM was generally defined and operationalized within referral criteria as three consecutive miscarriages in various maternity units/hospitals across the country, with some making exceptions in practice for women presenting with two miscarriages but who were older, had no living children, and/or had fertility issues. There was variation, however, with a need for standardization highlighted.

#### 3.1.1 The need for standardization in practice

Most health professionals stressed the need for, and benefits of, an agreed national definition of RM, with some noting, that different international guidelines were followed, for example, ESHRE or RCOG. Some women were also aware of the differing guidance (see Quote 1.1; Table 3). A few participants mentioned following national guidelines regarding how they defined RM (i.e., three consecutive miscarriages); some also noted how there was a move to two, internationally and within their own practice.

#### 3.1.2 Following the evidence: Who benefits from investigation and treatment/intervention

The definition of RM was framed within the context of referral criteria, with many health professionals, particularly those in tertiary care, stressing the need for these criteria to ensure that those perceived to benefit the most from the service would be seen/prioritized. They highlighted the challenging nature of RM, how investigations often did not provide any answers, and that women/couples have a good prognosis for future pregnancy without intervention. While it felt cruel and unfair to enforce strict criteria and/or refuse people for appointments, health professionals perceived that this was better than giving people false hopes, increasing stress levels, and potentially unnecessarily prolonging the time to pregnancy (see Quote 1.2; Table 3).

#### 3.1.3 Acknowledging complexity and need for flexibility: Considerations beyond the number of miscarriages

Several issues arose when discussing how RM is and/or should be defined. This extended beyond the number of miscarriages, to individual characteristics such as age, number of living children, fertility status, as well as circumstances related specifically to the miscarriages such as if they were consecutive or not, whether it was a biochemical pregnancy and the gestational age at the time of loss.

Clinicians and those in management roles generally felt that RM should be defined as three consecutive miscarriages, with exceptions made for those who were older (e.g., aged 35/38/39/40+) and/or did not have any living children or those with fertility issues—and that these should be considered after two miscarriages (see Quote 1.3; Table 3). This was also evident in referral criteria—which often stated three consecutive miscarriages, but in practice was more individualized, with the clinician generally holding the decision‐making power; a minority of whom stuck rigidly to the criteria, ‘adamant they will not investigate before three’ (S2) (see Quote 1.4; Table 3).

A few health professionals (including Clinical Midwife/Nurse Specialists, Public Health Nurses and General Practitioners) mentioned the need to consider cultural factors when considering age cut‐offs within the definition, such as cultures where women have, or are expected to have, children at younger ages.

Some health professionals and women and men with lived experience, however, thought that RM should be defined as two miscarriages, given the impact on women in terms of psychological/emotional impact and delaying potentially beneficial interventions (see Quotes 1.5 and 1.6; Table 3).

Many stressed the need for flexibility within the standardized definition, not to lose the ability to adopt a case‐by‐case approach when ‘it's very easy to write a guideline and pigeon‐hole everyone into the same kind of criteria, but it doesn't work that way especially, with this type of condition’ (OBGYN‐H3) (see Quote 1.7; Table 3).

Within discussions, consecutive miscarriages were generally referred to; however, some participants (both health professionals and women with lived experience) expressed concern with this criterion, and how women/couples can struggle with it, with some participants having been denied investigations on these grounds (see Quotes 1.8 and 1.9; Table 3).

A few health professionals also highlighted the need for miscarriages to be defined as losses arising from pregnancies confirmed by ultrasound. Other health professionals, however, stated that this did not factor into their practice and they ‘would just treat them all as having achieved a pregnancy that then didn't continue’ (OBGYN‐H2). Some women with lived experience (and General Practitioners) raised their concerns about the need for confirmation as they felt that both they and their miscarriages were questioned/dismissed/not acknowledged (see Quote 1.10; Table 3). A similar issue arose regarding the timing of a first‐trimester miscarriage. Several women and men with lived experience made the distinction between earlier and later first‐trimester miscarriages, and how it could be more difficult to wait until you had three miscarriages if they were later in gestation.

Some participants mentioned the inclusion/exclusion of ectopic and molar pregnancies within the definition of RM but there was little consensus. One participant mentioned that if ‘it's a new partner, that they might not meet the criteria. The criteria is not written down. But that's another frightening bit that they've mentioned in the past, and questioned in the past if the partner is different or … Whereas it shouldn't really’ (GN1).

#### 3.1.4 Limited resources constrain how RM is defined and/or how the definition is operationalized

Many health professionals, and women with experience of RM, acknowledged that costs and other resources impacted who could be seen/investigated for RM. This included staff running clinics (including Consultants and Clinical Midwife/Nurse Specialists) and clinics of duration and frequency to accommodate needs. A few clinicians mentioned that ideally, they would like to have genetic testing undertaken for all miscarriages; however, current resources did not permit this. Some General Practitioners noted that while they could conduct certain investigations within primary care; they could not access others, such as genetic testing. A few clinicians noted that despite having samples taken, laboratories refused to conduct analysis if women had not met the referral criteria, that is, experienced three consecutive miscarriages, thus creating a barrier at a further juncture in the system by gatekeepers.

Some clinicians felt that ‘it's probably a good compromise by keeping it at three for the return on the tests and investments that, you know, those patients are exposed to’ (OBGYN‐H2). Costs were often used to justify reasons for strict referral criteria during consultations with women/couples and in response to hospital complaints (see Quote 1.11; Table 3). While women and men with lived experience of RM appreciated these resource constraints, they felt that they could be better supported by the system (see Quote 1.12; Table 3).

### 3.2 THEME 2: The definition is a route to finding an answer, validating women/couples' experience of loss and providing necessary supports

This theme describes how the way that RM is defined holds significance for women/couples beyond entry to the system—it can be their route to finding a reason or meaning for their loss, and (potentially) realizing their dreams of becoming a parent, in addition to validating their loss and its significance. Some can, however, fall through the cracks in current service provision, existing in liminal spaces, particularly—though not exclusively—when RM is defined as three miscarriages.

#### 3.2.1 Looking for a reason/answer

All participants—those delivering/managing services and supports, and women and men with lived experience—spoke about the impact of RM, psychologically as well as physically, and how recurring miscarriages can increase anxiety. Women can also feel guilt, blame and a loss of confidence in womanhood. Feeling powerless, women and men search for answers (through health professionals, peers, social media/online research and elsewhere) as to why their multiple miscarriages happened, and what they can do, or can be done, to prevent them from happening if they wish to get pregnant again, sometimes waiting for answers before they embark on another pregnancy.

Many spoke about how investigations could provide reassurance (or sometimes false reassurance) because ‘[To] have a confirmed diagnosis it's almost like a relief’ (CMS4), but might not always provide an answer or ultimately the baby they long for. Many participants highlighted the negative impact that this can have, particularly if women/couples were older, and felt that time was going against them (see Quotes 2.1 and 2.2; Table 4). Many health professionals spoke about the need to manage women/couples' expectations; such discussions often took place within the context of supportive care (see Quote 2.3; Table 4).

#### 3.2.2 Feeling frustrated, or dismissed when you do not meet the criteria

Those delivering/managing services and supports, and women and men with lived experience, often spoke about women/couples feeling frustrated and dismissed when denied access to investigations and/or potential answers, particularly after two miscarriages. This was sometimes exacerbated during conversations in which clinicians highlighted how common miscarriage was and/or that it was often down to luck or chance. Such conversations often arose as part of efforts to (re)set expectations regarding the value of investigations and/or treatments and future prognosis, and reassure women/couples of the evidence‐based justification for their criteria. Both sides could feel this sense of powerlessness, citing the nature of RM (see Quotes 2.4 and 2.5; Table 4).

#### 3.2.3 Existing in a liminal space, falling between the cracks in service provision

Many participants highlighted the gap in service provision when RM was defined as three consecutive miscarriages; those who had experienced two miscarriages were often denied entry to the system for investigations, and not provided with any follow‐up and/or support—left ‘in limbo’ (PW10) (see Quotes 2.6 and 2.7; Table 4). A few mentioned that the definition of RM, or rather how it was operationalized as referral criteria, contributed to the provision of ‘a portal for support as well as, you know, obviously investigations and all that’ (CMS8); even though supportive (including bereavement) care was a key feature of current service provision, access to it was constrained by the referral criteria.

### 3.3 THEME 3: Working around the definition—Advocacy and impacts

Given issues regarding the definitions of RM in use, highlighted within Themes 1 and 2, Theme 3 describes a variety of advocacy efforts that were reported by participants such as working around the referral criteria themselves or through others and/or deferring to the private system; these were seen to have both positive and negative implications.

#### 3.3.1 Advocacy efforts: ‘To hell with the system’, ‘Just lie and say you've had three’ (Over‐riding the referral criteria)

Some health professionals spoke about advocating on behalf of women/couples who did not meet the specified referral criteria, or even circumventing the criteria to ensure that they had their needs met and that women/couples received the best care possible within a constrained system, that is, were seen and/or investigated. Examples included putting three instead of two miscarriages on referral forms, and one clinician also spoke about not mentioning that one of the three pregnancies was ectopic (see Quote 3.1; Table 5). This was very much appreciated by women/couples, who felt that both they and their needs were validated/recognized/appreciated (see Quote 3.2; Table 5).

Some women with experience of RM spoke about instances where they, or others, had been encouraged ‘Just lie and say you've had three’, often out of desperation (see Quote 3.3; Table 5). Some reported that health professionals—even they themselves—sometimes questioned women's reports of the number of miscarriages they had experienced as part of efforts to gain access to the system (see Quote 3.4; Table 5).

#### 3.3.2 Dealing with inappropriate referrals: ‘They still send in people, which is really cruel’

A few clinicians noted that such advocacy efforts might not always be in the best interests of women/couples and could do more harm than good by raising their expectations, putting them through unnecessary testing or delaying their time to pregnancy, even though denying a woman/couple of an appointment felt cruel and invalidation of their loss. One noted: ‘because it's such strict criteria to get referred, I think it prevents that [inappropriate referrals] happening … Just with the midwife being with the best of intentions being an advocate for the patient and trying to be helpful and trying to get someone into a clinic’. (OBGYN‐H4)

#### 3.3.3 ‘If it's not available in the public they will go to the private’

Many participants spoke about women/couples turning to, or being accommodated within, the private sector if they were denied entry to the public system, whether they could afford it or not. This sector was seen to have less strict criteria for investigations and/or more room for deviation from the rules, in addition to potentially providing unnecessary investigations and/or treatments (see Quote 3.5; Table 5).

Some health professionals (predominately Clinical Midwife Specialists and Sonographers) actively—but covertly—encouraged women/couples to seek private care (see Quote 3.6; Table 5). A few clinicians admitted to seeing women privately when they had been denied access to investigations within their care in the public system. One clinician who had a public and private practice stressed that they applied the same definition and criteria for both (despite negative reactions from colleagues in the private practice); however, some felt they could circumvent the public system (see Quote 3.7; Table 5).

## 4 DISCUSSION

In our study, we explored views of how RM is and/or should be defined from the perspectives of women and men with lived experience of RM and those involved in the delivery/management of services and supports. Findings from our 62 interviews highlight the importance of how the definition of RM is conceptualized and subsequently operationalized. In three interlinked themes, we found that a standardized definition of RM—one which considers research evidence, individual needs and healthcare resources—is needed. For women/couples who experience multiple first‐trimester miscarriages, how the condition is defined enables access to care. This offers the potential of finding a cause, the provision of support, and possible treatment, as well as validation of their experience or loss. Those who do not meet the criteria, however, can be denied access to a system of care and support, often resulting in advocacy to circumvent the referral criteria and/or seek care privately. This may have unintended consequences such as inappropriate referrals and unnecessary investigations and/or treatments.

Our findings reinforce the need to review and standardize how RM is defined, and for greater nuance around this, including how the definition is operationalized and services are organized. While definitions and recommendations within RM guidelines internationally vary,^2^ there is increasing support for offering certain investigations after two miscarriages^6^, ^8^, ^10^ and ongoing emphasis on providing individualized and supportive care for all women/couples.^10^ As evidenced in our study, defining RM is complex and considerations beyond the number of miscarriages are required. For example, issues regarding whether miscarriages were consecutive or not, whether a woman/couple had a living child or not, and the gestational age at which the miscarriage occurred, were all mentioned in the context of the definition and referral criteria. While some guidelines specify that miscarriages must be consecutive,^29^ others argue that women/couples should be managed the same, whether consecutive or not.^4^, ^6^, ^30^

It is important to emphasize that people will experience pregnancy loss differently and an individualized approach is needed.^20^ In line with previous research,^16^ we found that women/couples can experience grief and loss regardless of the gestation of the pregnancy loss and regardless of whether or not they have living children. In addition, although there is a body of evidence to support that greater maternal age and number of previous pregnancy losses are independently associated with increased miscarriage rates,^31^ it is important to note that age cut‐offs may not take into consideration cultural norms in expectations regarding the age at which women are expected to have children.^20^

How we define/diagnose conditions can result in stigmatization and exploitation^20^ and providing individualized care without normalizing or over‐medicalizing the condition is a key element of RM care.^10^, ^18^ In recent decades, there has been increased medicalization of early pregnancy loss, within a changing social and technical landscape.^32^, ^33^, ^34^ Clinicians in our study stressed the importance of not over‐medicalizing RM. They cited evidence‐informed reasons including that over half of RM cases are unexplained, and the limited evidence that treatments targeting perceived risk factors improve the prognosis for women/couples—which is good without intervention.^10^, ^18^, ^35^ Widening definitions of conditions can cause harm by exposing more patients to the adverse effects of treatments, triggering an investigation and prescribing cascades, increasing anxiety and placing a financial burden on patients and wider society.^36^ Such impacts can be overlooked in guideline development, however, as can conflicts of interest among guideline panel members.^37^ While clinicians felt that a definition of three was appropriate, other health professionals and women and men with lived experience felt that two would be more appropriate as women/couples can experience significant distress^1^, ^38^ and fall through the cracks in service provision.

Women and men with lived experience can strive for medicalization, even if this is not something that they would have favoured before their experience of RM, as we observed and as noted in the literature more broadly regarding pregnancy and childbirth.^39^, ^40^ In line with existing research, participants in our study expressed frustration when denied investigations following a second miscarriage, particularly in cases of advanced maternal age^16^, ^17^ and the lack of recognition of the psychological impacts of RM.^17^ Women/couples seek answers or explanations from health professionals which can lay a path for what happens next.^15^ Health professionals are gatekeepers of RM services, applying definitions and referral criteria.^20^ As noted by Coomarasamy et al.,^10^ some women/couples in our study sought out many different health professionals/services in their search for a cause. Finding a medical explanation for miscarriage may reduce feelings of guilt and self‐blame, including the questioning of womanhood, as part of strategies to regain some sense of control.^16^, ^17^, ^18^, ^41^, ^42^ While an unexplained miscarriage can be a relief for some,^16^ for others it can provide little reassurance given the lack of scientific/medical explanation that society has come to expect.^34^ Similar to that observed by van den Berg et al.,^15^ women and men with lived experience in our study felt frustrated and dismissed when health professionals highlighted how common miscarriage was and/or that it was often down to luck or chance, often in the context of them not meeting referral criteria for clinics.

In the Republic of Ireland, RM care is delivered within the 19 public maternity units/hospitals. Access to publicly funded care is dependent on meeting certain referral criteria, often three consecutive miscarriages with some derogations, as highlighted by participants. Otherwise, as we observed, women/couples can turn to private providers, with associated financial costs. Internationally, there is limited research on the financial costs that women/couples with RM bear.^1^ Within international guidelines, there is also a lack of consideration of the potential resource implications of applying recommendations.^2^ As observed in other health areas, actions that staff take when seeking to compensate for perceived inadequacies in service provision can have many consequences; some positive (e.g., receipt of services/care for some patients, protection/relief from moral injury for health professionals) and some negative (e.g., inequity and exclusion).^43^ Some health professionals spoke of moral dilemmas and the hidden practices they engaged in to assist women/couples to surmount referral criteria. Equity considerations are important and are particularly relevant within the Irish context at present as the State endeavours to implement universal health and social system where everyone has equal access to services based on need, and not the ability to pay.^44^, ^45^

Strengths of our study include drawing on the views and experiences of multiple participant groups (women and men with lived experience, and those involved in the delivery/management of services and supports) and researchers (e.g., in data collection and analysis) to capture a deeper, more complex, multifaceted understanding of how RM is defined.^46^ To further enhance the credibility of the work, we incorporated member reflections via feedback from our Research Advisory Group members and use ‘thick description’ with illustrative quotes throughout to facilitate reader interpretation. The latter also enhances the rigour of the work, in addition to a detailed description of our data collection and analysis procedures.^46^ Important information to consider when judging the transferability of findings, is that this study was conducted in the Republic of Ireland, where there is currently no RM guideline and RM is generally defined as three consecutive miscarriages. Furthermore, while people involved in the delivery/management of services and support spoke about their experiences and views of the topic in relation to people of varying age, ethnicity and sex/gender, people with lived experience of RM who participated in our study were typically white Irish and heterosexual; however, we did not collect detailed demographic information.

Our findings and analysis encourage a more contextualized overview of how RM is defined than previous work has afforded by providing in‐depth insights into the views of women and men with lived experience, and those delivering/managing services and supports. A more nuanced approach to defining RM is warranted, one which is evidence‐informed and recognizes the needs of women/couples. Our findings reinforce international calls for standardization, and a graded approach to miscarriage care in which women/couples are offered appropriate, individualized, support following one, two and three or more miscarriages.

## AUTHOR CONTRIBUTIONS

**Rebecca Dennehy**: Conceptualization; methodology; investigation; data curation; formal analysis; project administration; writing – review & editing. **Marita Hennessy**: Conceptualization; methodology; investigation; data curation; formal analysis; project administration; writing – original draft; writing – review & editing. **Sarah Meaney**: Conceptualization; methodology; funding acquisition; formal analysis; supervision; writing – review & editing. **Karen Matvienko‐Sikar**: Formal analysis; writing – review & editing. **Ria O'Sullivan‐Lago**: Formal analysis; writing – review & editing. **Jennifer Uí Dhubhgain**: Methodology; formal analysis; writing – review & editing. **Con Lucey**: Methodology; formal analysis; writing – review & editing. **Keelin O'Donoghue**: Conceptualization; methodology; funding acquisition; formal analysis; supervision; writing – review & editing. All authors approved the final version of the manuscript.

## CONFLICTS OF INTEREST

R. O. S. is employed by Waterstone Clinic, a private fertility clinic operating within the Republic of Ireland. J. U. D. is Secretary of the Miscarriage Association of Ireland; K. O. D. is Clinical Lead, Guideline Development, National Women and Infant's Health Programme, Health Services Executive, Ireland; M. H. is a member of the Expert Advisory Group for the National Maternity and Gynaecology Clinical Guideline Programme and a member of the Guideline Development Group for the Clinical Practical Guideline on Recurrent Pregnancy Loss, both under the National Women and Infant's Health Programme, Health Services Executive, Ireland and Institute of Obstetricians and Gynaecologists, Royal College of Physicians of Ireland—none of these roles involves advocating for any particular definition of RM. The remaining authors declare no conflict of interest.

## ETHICS STATEMENT

Ethical approval for the study—‘Exploring stakeholder views on RM services in the Republic of Ireland’—was granted by the Clinical Research Ethics Committee of the Cork Teaching Hospitals (CREC) [Reference number: ECM 4 (ff) 10/03/2020 & ECM 3 (gggg) 09/04/2020].

## Supporting information

Supplementary information.

## Data Availability

While data were pseudo‐anonymized for the purposes of this study, participants are still potentially identifiable from transcripts due to their particular roles, lived experiences, and/or turns of phrase. We are not, therefore, sharing the data from this study; this is in line with the ethical approval granted, including consent processes.
